# Expanding the conversation: A Person-centred Communication Enhancement Model

**DOI:** 10.1177/14713012221080252

**Published:** 2022-04-24

**Authors:** Deanne J O’Rourke, Michelle M Lobchuk, Genevieve N Thompson, Christina Lengyel

**Affiliations:** College of Nursing, University of Manitoba, 423134 Rady Faculty of Health Sciences, Winnipeg, MB, Canada; Department of Food and Human Nutritional Sciences, 8664 University of Manitoba, Winnipeg, MB, Canada

**Keywords:** long term care, dementia care, person centred care, person centred communication, theory

## Abstract

The intricacy and impact of human communication has long captured the attention of philosophers, scholars and practitioners. Within the realm of care and service provision, efforts to maximize outcomes through optimal person-provider communication have drawn research and clinical focus to this area for several decades. With the dawning of the person-centred care movement within healthcare, and in particular long-term care home and dementia care settings, improvement in care providers’ use of person-centred communication strategies and enhancement of relationships between residents, their families and care providers are desired outcomes. Thus, several person-centred care and communication theoretical perspectives have been employed to ground study in this field. However, a comprehensive theoretical position to underpin person-centred communication in dementia and older adult research does not exist to our knowledge. To offer expansion to the theoretical work in this emerging field, a Person-Centred Communication Enhancement Model for long-term care and dementia care is proposed, as well as rationale for its development. This discussion will also provide an overview and critique of the extant philosophies, theories, frameworks and models that have been utilized in the study of person-centred communication within the context of long-term care and dementia care.

## Introduction

‘All real living is meeting’.

This quote from Martin Buber’s philosophical writings on personal relationships (Buber, 1958, p. 11) imbues the significance of our interconnectedness with the world. *Meeting* in this context does not refer to formal committees or meetings, but instead represents an awareness, openness, and acceptance of the experience of being in the world and living within the context of human relationships (Buber, 1958). Although person-centred communication can and is employed in a variety of personal, social and healthcare settings, this discussion will relate specifically to its use in the context of long-term care homes. As many individuals in long-term care homes experience dementia, a successful person-centred communication approach in this setting must encompass strategies to communicate effectively with people with dementia. In alignment with the importance Buber places on relationships and interconnectedness with other humans, person-centred communication aims to enhance interactions and relationships between residents/families and care providers within the long-term care homes setting. With the impact of the COVID-19 pandemic and resultant isolation and reduced contact between long-term care home residents and their family and friends, promoting human connectedness between care providers and residents is of utmost importance within this recent context. It is from this vantage point we discuss the expansion of theoretical perspectives relative to the study of person-centred communication within the context of long-term care homes.

Discussed more fulsomely below, the central attributes of a person-centred care philosophy, namely relationship, individualism, holism, respect and empowerment, are ideally integrated into the care milieu through day-to-day communication and interactions between providers and residents (McCormack & McCance, 2006; Nolan, Davies, & Brown, 2006; O’Connell, Ostasziewicz, Sukkar & Plymat, 2008). Research indicates that residents in long-term care homes react more positively (Savundranayagam, Sibalija, & Scotchmer, 2016), experience enhanced mood and affect (McGilton, Sidiani, Boscart, Guruge, & Brown, 2012) and report higher levels of well-being (Custers, Kuin, Risken-Walraven, & Westerhof, 2011) when providers demonstrate effective relational (person-centred) behaviours during interactions. Conversely, it has been suggested that missed opportunities for person-centred communication in long-term care homes undermine provider–resident communication and relationships and can result in negative responses from residents, including resistiveness to care and distress (Savundranayagam et al., 2016).

Over the past decade, empirical research pertaining to knowledge expansion and application of person-centred communication approaches has steadily increased. Although certain key theoretical elements of effective interpersonal communication within the context of dementia have been identified, such as mutuality, personhood and anxiety reduction (Ennis, Mansell, McEvoy, & Tai, 2019), a comprehensive theory or model to support evolving person-centred communication applied research in long-term care homes is lacking. As such, a collective examination and critical analysis of the theoretical perspectives employed in person-centred communication research was undertaken to provide insight into the contributions of these viewpoints to this field of study, as well as address evident theoretical gaps within an expanded model.

This discussion will begin with a review of theoretical foundations, components and linkages between person-centred care and person-centred communication. Based on extant theoretical works and person-centred communication outcomes observed to date, a Person-Centred Communication Enhancement Model (PC-CEM) will be presented. Lastly, a critique of existing person-centred care and person-centred communication theoretical perspectives will be provided to demonstrate gaps in the current theoretical literature that can be addressed by the PC-CEM.

## Person-centred care and person-centred communication

### Conceptual considerations

Person-centred care is a concept that has been promoted widely as the gold-standard approach for care provision and has gained a moral authority as ‘just the right thing to do’ (Duggan, Geller, Cooper, & Beach, 2006, p. 272). Kitwood’s (1997) foundational work on person-centred care, personhood and relationships established a theoretical basis for how person-centred dementia care is defined. Within a person-centred approach, the person is the focus of the care or encounter, and all interactions and interventions are planned and delivered within context of that central aim (Harding, Wait, & Scrutton, 2015).

Person-centred communication is felt to be a necessary operational component of person-centred dementia care (Brooker, 2007; Downs & Collins, 2015; Kitwood, 1997). As such, efforts have been made to define the theoretical elements of person-centred communication within older adult and dementia care contexts. Research has indicated that effective person-centred communication in dementia care consists of both linguistic (language-based) strategies and relational (person-centred) approaches (Ryan, Byrne, Spykerman, & Orange, 2005; Ryan, Meredith, MacLean, & Orange, 1995; Savundrananyagam & Moore-Nielsen, 2015). The linguistic elements of person-centred dementia communication are defined as language-based strategies that promote communication goals of reciprocity, clarity/coherence and continuity when communicating with individuals with dementia. The relational aspects of person-centred dementia communication include approaches that extend beyond the functional aspects of communication to address personhood, inclusive of the individual’s life history, values and preferences (Savundrananyagam & Moore-Nielsen, 2015).

### Theoretical linkages

In examining existing person-centred care and communication theoretical works relative to person-centred communication, novel linkages between these two areas are evident. Firstly, the underlying theme that interconnects person-centred care and person-centred communication is the desire to achieve a relational approach to interpersonal interactions and care. Within person-centred approaches, it is through relationships with others that recognition and respect for the person and preservation of their personhood is realized (Brooker, 2007; Kitwood, 1997). Sabat’s research (2002) pertaining to manifestations of selfhood in the context of dementia suggests that continued existence of the social personae ‘self’, which encompasses various social roles developed over one’s lifespan, is dependent upon the cooperation of and engagement with others. The person-centred theoretical work indicates that communication approaches enacted by providers offer a critical pathway to developing and maintaining meaningful person-provider relationships and supporting person-centred care (McCormack & McCance, 2006). The literature also suggests that individuals with moderate dementia can engage in meaningful communication and mutually satisfying relationships. In many cases, they are also able to evaluate social behaviour and interpret the difference between ‘task-based’ and ‘person-centred’ behaviour (Sabat & Lee, 2011). Thus, a connection to the person-centred care theoretical literature is essential to underpin the elements of person-centred communication approaches.

Secondly, the cohesive theoretical principles of person-centred care, namely valuing and respecting the person, and perceiving them as a unique individual with specific needs and care approaches (Brooker, 2007; Kitwood, 1997) are reflected with the elements of person-centred communication. Effective language-based accommodation strategies plus these relational (person-centred) approaches are necessary to ensure respectful communication and quality interpersonal relations whilst promoting or maintaining communication competence (Savundranayagam & Moore-Nielsen, 2015) and avoiding the theorized communication predictions of ageing and dementia, as discussed below (Ryan et al., 1986).

Thirdly, the concepts of person-centred care and person-centred communication also share similar outcomes including enhanced psychological well-being and quality of life, and increased satisfaction with person-provider relationships for the individual living in a long-term care home. For the provider, common indicators also exist across both theoretical fields and include increased knowledge and competence, enhancement of the quality of relationships with residents, and increased work satisfaction. Both concepts also reflect a critical epistemology in that they seek to promote action and change by the creation of person-centred therapeutic care paradigms and positively influence individual and societal views in relation to ageing and dementia.

Despite this theoretical alignment, a single, stand-alone framework, theory or model that fulsomely incorporates both person-centred principles, the specific relational and linguistic elements, outcomes of person-centred communication in long-term care home or dementia care settings and contextual considerations does not currently exist in the literature. This gap could be addressed by amalgamating existing theoretical perspectives to provide a template to support all aspects of effective person-centred dementia communication. In addition, specific individual, provider- and system-level outcomes based on evolving empirical research would also need to be reflected in an enhanced theoretical perspective to support the study of person-centred communication in long-term care homes and dementia care.

## The person-centred communication enhancement model (PC-CEM)

**Figure 1.**
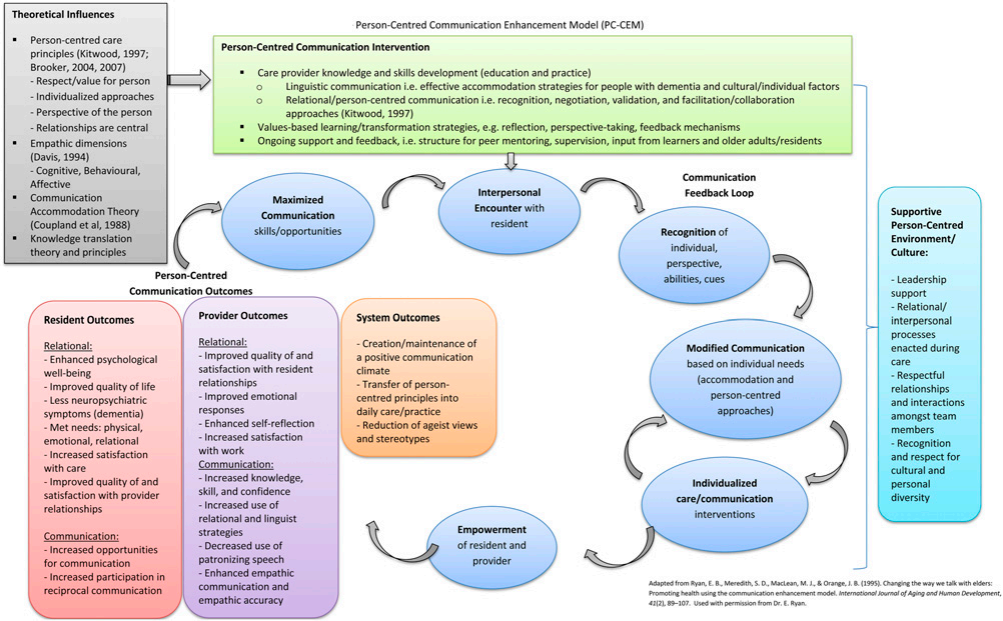
Person-centred communication enhancement model (PC-CEM).

As such, the Person-Centred Communication Enhancement Model (PC-CEM) with applicability to the long-term care home/dementia care setting (Figure 1) is proposed. Adapted from Ryan and colleagues’ (1995) Communication Enhancement Model, the PC-CEM offers additional clarity and specificity surrounding the elements and outcomes of person-centred communication within the context of long-term care and dementia care, as well as theoretical and practical considerations to inform a person-centred communication intervention. The PC-CEM also identifies current gaps and areas for future theoretical development and research. A fulsome description of the model’s components and application opportunities is discussed below.

### Person-centred communication intervention

The person-centred communication intervention, as outlined at the onset of the PC-CEM, consists of evidence-informed components found to be associated with effective implementation and outcomes (Figure 1 – box top centre). These include care provider knowledge and skills development through education and practice, and value-based learning and transformation strategies.

#### Knowledge and skills development

The extant literature suggests that providers’ knowledge and skills development are supported through training/education and practice opportunities within real-world settings (McGilton et al., 2009). Also, as person-centred communication is comprised of linguistic, relational and personal elements, all aspects should be addressed within the intervention components. Examples of linguistic communication skills could include effective accommodation strategies for older adults, as well as dementia-related communication strategies (verbal and non-verbal) and cultural/individual influences on communication. Providers’ skill development regarding relational aspects of person-centred communication would involve imparting knowledge, awareness and recognition of person-centred elements of communication (i.e. recognition, negotiation, validation and facilitation) as outlined by Kitwood (1997).

#### Values-based learning

To facilitate a shift in caring culture to a more person-centred approach, person-centred communication interventions would also need to incorporate values-based learning and transformation strategies (Viau-Guay et al., 2013), such as self-reflection, perspective-taking and performance feedback. Additionally, a person-centred communication intervention should include an ongoing mechanism for support and feedback, using strategies such as peer mentoring or supervision (Aubry et al., 2013). Input should be gathered from learners, older adults in long-term care homes and people with dementia on an ongoing basis as to needed adjustments to the communication approaches.

### Theoretical influences

Providing an overarching framework, relevant theoretical perspectives act to inform all aspects of the person-centred communication approach and intervention (Figure 1 – box upper left). The person-centred care principles of respect and value for each person, an individualized approach to care and service, and recognition of the centrality of relationships within the long-term care home context and caring culture (Brooker, 2004; 2007; Kitwood, 1997) offer a consistent lens in which to view communication interventions, interactions and outcomes. Additionally, the inclusion of empathy theory and consideration of the cognitive, behavioural and affective empathic dimensions (Davis, 1994) provides a basis to explore and establish theoretical and empirical linkages between empathy and person-centred communication.

Furthermore, a person-centred communication intervention should consider theoretical implications in relation to the social and personal context of communication. Coupland and colleagues’ (1988) Communication Accommodation Theory, described in further detail below, suggests that communication should be viewed from a dual socio-linguist perspective. The theory purports that communicators modify or adjust their speech and non-verbal behaviours based on individual values, motivations and perceptions of the other person’s capabilities. Thus, an effective communication strategy should also acknowledge these social considerations, as well as potential biases a communicator may have when conversing with an older person or a person with dementia. This theoretical perspective has a strong linkage to empathy theory mentioned above, as a key aspect of cognitive empathy or perspective-taking is being aware of self first before engaging in the other person orientation (Davis, 1994).

Lastly, application of knowledge translation theory and principles have been associated with effective transition of knowledge into action (Colquhoun, et al., 2017; Williams, Perkhounkova, & Bossen, 2016). Factors to consider when integrating a new intervention into practice include identification of barriers, linkage of barriers to selection of the intervention components, and seeking input from users as to feasibility and acceptability of the intervention (Colquhoun et al., 2017).

### Communication feedback loop

In consideration of an actual person-centred communication exchange between a provider and resident, the PC-CEM follows the same cyclical progression as the Communication Enhancement Model (Ryan et al., 1995) and begins with a provider’s *interpersonal encounter* with a long-term care resident (Figure 1 – ovals). As a result of the person-centred communication intervention, the provider enters the interpersonal encounter with enhanced knowledge and awareness of person-centred values and person-centred communication (linguistic and relational) strategies. Based on initial communication attempts by the provider and knowledge of the person, there is *recognition* of the individual resident’s perspectives, abilities and communication cues. As a result of this communication feedback, the provider *modifies their communication* approaches (i.e. appropriate linguistic accommodation and person-centred approaches) based on the resident’s needs and abilities. Ongoing exchanges may assist the provider to further tailor communication approaches based upon their assessment of the resident’s response. Through improved communication and mutual participation in conversation, *individualized care and communication interventions* can be developed and implemented. Successively, as a result of this mutual exchange and partnership approach to the caring relationship, resident and provider *empowerment* occurs and results in *positive outcomes* for both communication partners. These positive outcomes are hypothesized to *maximize communication* skills and opportunities for both resident and provider, offering enhanced confidence and knowledge to bring forth in future encounters (Ryan et al., 1995).

### Person-centred communication outcomes

Based on research evidence, it is hypothesized that person-centred communication could result in specific relational and communication outcomes for the resident and provider, and the system context overall.

#### Resident outcomes

Relational outcomes of person-centred communication approaches pertaining to residents are outlined in Figure 1 (rectangular box lower left). These include the potential for enhanced psychological well-being and improved quality of life (McGilton et al., 2016, 2017), a reduction in neuropsychiatric symptoms for people with dementia (McGilton et al., 2017), holistic meeting of physical, emotional and relational needs, increased satisfaction with care (Grosch et al., 2008; McGilton et al., 2017), and improved quality of and satisfaction with provider relationships (McGilton et al., 2010). Outcomes pertaining to communication enhancement on the part of the resident are also noted in the literature. These include increased opportunities for communication and participation in reciprocal communication (i.e. mutual exchange of dialogue) (McGilton et al., 2010, 2017) and confidence in their own communication skills (McGilton et al., 2017).

#### Provider outcomes

Provider relational outcomes have also been reported (Figure 1 – rectangular box lower left). These include improved quality of and satisfaction with resident relationships (Coleman & Medvene, 2013), improved emotional responses (McGilton et al., 2010), an enhanced ability to engage in self-reflection (James, Phillipson, McCrossan, & Falck 2013; James, Hall, Lombardo, & McGovern, 2016), and increased satisfaction with their work (McGilton et al., 2016). Outcomes related to improved provider communication skills are also noted in the literature. These are in relation to increased communication knowledge and skill (McGilton et al., 2010; Passalacqua & Harwood, 2012), increased use of relational and linguistic accommodation strategies (Barbosa et al., 2016; Passalacqua & Harwood, 2012), decreased use of patronizing speech (Williams, Herman, Gajweski, & Wilson, 2009; Williams et al., 2016), and enhanced empathic communication and accuracy (Lobchuk et al., 2016; 2018).

#### System outcomes

It is also proposed that the person-centred communication approach has implications at the macro level. Possible system outcomes (Figure 1 – rectangular box lower left) include translation of person-centred care principles into the culture of daily care and practice (Grosch et al., 2008), creation and maintenance of a positive communication climate, and a reduction of ageist views and stereotypes pertaining to long-term care residents and people with dementia (Dewar & Nolan, 2013; Kagan et al., 2008; Ryan et al., 1986; 1995).

### Supportive person-centred environment/culture

The final component of the PC-CEM parallels the Communication Enhancement Model (Ryan et al., 1995) in that efforts to enhance person-centred communication skills and opportunities, and the subsequent exchanges that ensue between providers and residents, occur within the context of *multiple environmental influences* (Figure 1 – rectangular box far right). A supportive person-centred environment and culture are considered crucial ingredients to successful person-centred communication. Critical components consist of leadership support, inclusion of relational/interpersonal engagement during daily care activities, respectful relationships and interactions among team members, and recognition and respect for cultural and personal diversity (Li & Porock, 2014; Squires et al., 2015).

In summary, as an expansion of the Communication Enhancement Model (Ryan et al., 1995), the PC-CEM for use in long-term care homes and dementia care offers a theoretical basis for the formulation of research inquiries specific to person-centred communication approaches and interventions. The PC-CEM has theoretical flexibility to broadly support person-centred communication in general or more specific person-centred dementia communication. The model includes parameters to consider in respect to the design and delivery of an evidence-informed person-centred communication interventions and contextual factors to facilitate successful integration into practice. The feedback loop supports the components of a person-centred approach to communication where an individual’s perspective and abilities are considered, and adjustments made as necessary. The enhanced model delineates outcomes at the resident-, provider- and system-levels based on empirical findings to date.

## Implications and significance

Person-centred communication approaches have the potential to weave person-centred principles into the fabric of everyday practice and enhance outcomes within a care or service delivery setting. As a result of person-centred communication approaches enacted during day-to-day care and interactions, evidence suggests that long-term care home residents may experience enhanced quality of life, care outcomes, and satisfaction with care and their relationships with providers. Additional clarity in respect to the theoretical basis of person-centred communication is intended to promote a deeper understanding of pathways to promote stronger communication and enhancement of relationships between providers and residents in long-term care home/dementia care settings.

Although a focus of this theoretical discussion is to consider person-centred communication approaches applicable to residents in long-term care homes with dementia, the principles outlined in the PC-CEM could also apply to individuals in long-term care homes who do not have dementia. Specifically, a person-centred communication approach in these instances would focus more so on the person-centred (relational) and social elements of communication as opposed to the use of the linguist strategies applicable when communicating with a person with dementia. During a provider-person interaction, the communication feedback loop would be applicable as described above with the communicator adjusting their approaches based on assessment of the resident’s/older adult’s abilities and feedback from their conversation. Lastly, apart from decreased neuropsychiatric symptoms associated with dementia, the resident outcomes noted in the PC-CEM would be applicable to a long-term care home resident who does not have dementia.

The PC-CEM offers further theoretical guidance to support the translation of person-centred communication principles into practice. As a practical example, the PC-CEM was used to guide a study that pilot tested a video feedback intervention to enhance person-centred dementia communication skills of care aides working in long-term care (publication pending). The model provided the theoretical foundation for the development of the components of the person-centred dementia communication intervention, as well as offered guidance in the selection of care provider outcomes.

As the PC-CEM is intended to be fluid and expandable, there is an opportunity to test and refine its components and antecedents, explore new linkages within the model (i.e. person-centred communication and empathic dimensions), and include additional resident-, provider- and system-level outcomes as realized through further research in the field. There is also a potential for the model to be adapted to other settings and communication partners beyond the long-term care home context. As an example, Small and Perry’s (2012) Training in Communication Enhancement for Dementia (TRACED) program educates family members on person-centred communication compensatory (linguistic) and connecting (relational) strategies when communicating with a person with dementia living in the community. In this context, the PC-CEM could offer theoretical support for the components of the intervention itself, as well as sustainability strategies. The model’s communication feedback loop could represent an exchange between a family member and person with dementia. The outcomes in the feedback loop could be adjusted to reflect those established in the literature and others to be explored within the community/home setting. Lastly, overarching supportive person-centred environment and cultural factors could also be modified to reflect the home and broader community context.

## Critique and gaps in current theoretical perspectives

Upon review of the literature, a variety of theoretical perspectives have previously guided the study of person-centred communication within long-term care home and dementia care settings; however, a fulsome theory that supported the components of person-centred dementia communication was not evident. Thus, the following provides an overview of these extent theoretical perspectives, their contributions to this field of research and evident gaps when considered individually and collectively.

Of note, within this area of study certain philosophies, frameworks, theories and models have been used to underpin person-centred communication research. These terms are often conflated and used interchangeably; therefore, to preface this discussion some definitions are provided. A *philosophy* is a set of generalized views of the world that inform our understanding and beliefs and guide our actions. Philosophy provides the basis of theoretical thought and a foundation in which to design and conduct research. A (conceptual) *framework* is more refined than a philosophy, identifying variables and relationships among them to explain a phenomenon and set the stage for theory development. Grounded within a framework, a *theory* provides further clarity by outlining coherent relationships (often directional in nature), hypotheses and co-variables. Lastly, *models* have the narrowest focus and are developed to make assumptions about a specific set of parameters and variables. These are then tested against specific outcomes (Carpiano, 2006). However, what is felt to be most essential to consider is how a theoretical application is, or can be used, to ground a research study (Green, 2014). Thus, for the purposes of this review, the term *theoretical perspective* will be used to examine the contributions of the following theoretical positions that have been used to study person-centred communication within long-term care home and dementia care settings.

### Data sources

This discussion is based upon a search and review of relevant literature. The review was undertaken July 2018–March 2020 and included articles, books and dissertations from database inception in the SCOPUS, CINAHL and MEDLINE databases. Abstracts, titles and keywords were searched using terms relative to person-centred care/communication and theory (Table 1). The reference lists of relevant data sources were also hand-searched for additional references. Theoretical perspectives that addressed either person-centred care or person-centred communication and were applicable to person-centred communication approaches in long-term care home settings, older adults or people living with dementia were included in this summary. Theoretical perspectives that have been developed to foster person-centred care and approaches will be initially discussed, followed by those that have arisen from the communication field of study.

**Table 1.** Database search terms.

Concept	Search terms
Person-centred care/communication	‘person centred care’ OR ‘person centered care’ OR ‘partnership centered care’ OR ‘partnership centered care’ OR ‘patient centered care’ OR ‘patient centred care’ OR ‘client centred care’ OR ‘client centered care’ OR ‘resident centred care’ OR ‘resident centered care’ OR ‘relationship centred care’ OR ‘relationship centered care’ OR ‘family centred’ OR ‘family centered’ OR dementia OR communication
Theory	theory OR philosophy OR framework OR model

### Person-centred care theoretical perspectives

The review of the extant theoretical literature reveals a scholarly evolution of the articulation of person-centred and relational care. An overview of the assumptions, key elements and outcomes of these theoretical perspectives in order of their appearance in the literature is provided in Table 2. Seminal thought in relation to person-centred care began with broader philosophical and conceptual works (i.e. Brooker, 2004; 2007; Buber, 1958; Kitwood, 1993; 1997; Kitwood & Bredin, 1992) and expanded to further development of more refined theory and action-oriented models (i.e. McCormack & McCance, 2006; McGilton et al., 2012; Nolan et al., 2006; O’Connell et al., 2008; Røsvik et al., 2011) to guide implementation of person-centred principles into care and practice. In consideration of these theoretical perspectives, some consistencies and divergences are noted in respect to their key elements, assumptions and outcomes.

**Table 2.** Summary of person-centred care theoretical perspectives.

Author(s) Discipline Location	Theoretical perspective	Assumptions/Key elements	Outcomes
Buber, 1923 (original translation); 1958 (2nd Ed.) Philosophy; theology Austria	Buberian Social Existentialist Philosophy	•No I or singular person as humans are always in relation to the world and others •Two forms of relationships with others: I-It and I-Thou •I-It implies objectification of and detachment from the other; I-Thou involves involvement and investment in the other as a whole person	•I-Thou relationship: the only pathway to realize meaningful relationships with others
Kitwood and Bredin, 1992; Kitwood, 1993; 1997 Psychology UK	Person-centred Dementia care Philosophy	•Preservation of personhood in dementia; at risk for loss of personhood due to biomedical perspective •Recognition and respect for personhood occurs within the context of social relationships (established link to Buber’s I-Thou relationship) •Malignant social psychology: actions and attitudes of others that undermine personhood •Positive person work: affirming interactions that promote personhood	Three levels of outcomes •Individual-level: enhanced well-being and addressed psychosocial and relational needs in addition to physical ones •Provider-level: valued and respected as people with unique needs and feelings; also benefit from fulsome relationships •System-level: transformation away from a disease-oriented culture of care
Brooker, 2004; 2007 Psychology UK Røsvik et al., 2011 Norway	VIPS Framework VIPS Practice Model	•Contains 24 indicators of person-centred dementia care that can be used to assess and operationalize the VIPS concept oValue the person oIndividualized approach oPerspective of the person with dementia oSupportive social environment •Practical guidelines to provide a structure for application of VIPS in practice •Comprised of oStructured teamwork (roles and functions, practical knowledge, consensus meetings) oSupervision and training oSupportive management	Three levels of outcomes for the VIPS approach •Individual-level: improved care and services (e.g. decreased neuropsychiatric symptoms) •Provider-level: support daily application of person-centred care in a practical sense •System-level: Build implementation support for cultural person-centred transformation
McCormack & McCance, 2006 Nursing UK	Person-centred nursing Framework	•Comprised of four constructs 1) pre-requisites 2) care environment 3) person-centred processes 4) expected outcomes •Relationship between constructs: must achieve outer layers (i.e. constructs 1 through 3 above) to achieve outcomes situated in the centre of the framework •Applicable to all care settings and domains	•Individual-level: satisfaction with care, involvement with care, feeling of well-being •Provider and system/organization: creating a therapeutic culture
Nolan et al., 2006 UK	Senses Framework	•Emphasizes relationship-focused care or the impact of experiences of all participants, not only the person-provider •6 senses (security, continuity, belonging, purpose, achievement, significance) •To achieve high quality care, all individuals must experience relationships that promote these senses	•No explicit outcomes •Framework has been used to examine key care and service outcomes and how to create positive relationships
O’Connell et al., 2008 Nursing Australia	Tri-Focal Model of care	•Emphasis on partnership-centred care (resident/families, staff, service providers and students) •Three components: Partnership-centred care, positive work environment and evidence-informed practice for long-term care •Teaching nursing home concept fosters an effective learning and working environment	•Residents: improved quality of life and care (empowerment, knowing the resident, enhanced communication) •Staff: enhanced communication, improved work satisfaction, increased skills in evidence-based care, desire for further professional development
McGilton et al., 2012 Nursing Canada	Person-centred Framework in long-term care	•Structures: Nurse competences that include leadership qualities osupervision omentoring ocommunication ofacilitation •Processes: REAP framework oRelate well oEnvironmental manipulation oAbilities-focused care oPersonhood	•Resident-level: identification of met needs, reduction in responsive behaviours, creation of meaningful moments, enhancement of relationships •Provider-level: increased meaningful relationships, effective Registered nurse-care aide relations, increased work satisfaction

#### Consistencies

All perspectives are based upon the assumption that our human experience, purpose and value are grounded in social relationships. Further, meaningful relationships between individuals and providers support person-centred care by promoting an emotional connection, reciprocity and mutual benefit. Aligned key elements of the theoretical perspectives include respect and value for the person and their personhood, as well as supporting an individualized approach to the identification and provision of care needs by knowing the person. Most of the theoretical perspectives address outcomes at individual-, provider- and system-levels. Common individual-level outcomes include enhanced psychological well-being and quality of life, and satisfaction with all care needs, including emotional and relational, being met. Theorized provider-level outcomes include enhanced relationships with those in care, increased knowledge and skills in person-centred care and increased work satisfaction. All perspectives anticipate system-level improvements that support a positive transformation to a therapeutic person-centred care culture. Although only three pertain specifically to person-centred dementia care (i.e. Kitwood’s philosophy, the VIPS framework and the VIPS Practice Model), all appear applicable to the study of person-centred care in dementia and long-term care.

#### Divergences

In view of the inconsistencies between perspectives, some language and conceptual differences are evident. The historical development of person-centred theoretical work in the dementia care and long-term care setting reveals the use of different terminology, beginning with person-centred care (Kitwood, 1997; Brooker, 2004), with later works referencing relationship-centred care (Nolan et al., 2006) and partnership-centred care (O’Connell et al., 2008). Additionally, due to the evolution of the concept over time, earlier theoretical perspectives focused mainly on the person-provider relationship (Buber, 1958; Kitwood, 1997; McCormack & McCance, 2006), whereas further works have expanded upon the original intent to include other relationships within the care environment (Nolan et al., 2006; O’Connell et al., 2008). Lastly, not all the theoretical perspectives address contextual facilitators of person-centred care (Brooker 2004; 2007; Buber, 1958; Kitwood, 1997). However, those that recognize the need to provide sufficient environmental support note that necessary facilitators include a supportive leadership and work culture, evidence-informed knowledge, competencies of person-centred care and interpersonal processes enacted during care (McCormack & McCance, 2006; Nolan et al., 2006; O’Connell et al., 2008).

#### Limitations

In final consideration of the collective person-centred care theoretical perspectives, some limitations are evident. Firstly, due to the relative infancy of this field of theoretical development, apart from the Person-Centred Nursing Framework (McCormack & McCance, 2006), the majority of the perspectives have not yet been fulsomely tested or used to underpin empirical work. Secondly, although some dementia-related outcomes are offered within a few of the theoretical perspectives, appropriate measurement approaches for certain indicators need further elaboration for this population (e.g. satisfaction with care and meeting psychosocial needs in persons with dementia). Finally, most of these theoretical perspectives do not offer a specific theoretical linkage to person-centred communication in dementia. As Kitwood’s (1997) person-centred dementia care philosophy appears to be the exception (Ennis et al., 2019), Kitwood’s theoretical writings will be examined in further detail.

#### Kitwood’s person-centred care philosophy

Tom Kitwood (1937–1998), a British academic psychologist, began to articulate his ground-breaking philosophy in personhood and person-centred dementia care in the early 1990s (Kitwood, 1993; Kitwood & Bredin, 1992). Radical to the thinking at the time, his work sought to bring the elements of dementia-related neuropsychology and social psychology together into a single frame (Kitwood, 1997). This sparked a reconsideration of our understanding and perceptions of dementia that began over two decades ago and continues to represent the current ideal for quality dementia care services (Brooker, 2004; 2007; Mitchell & Agnelli, 2015). His work has also provided a platform from which subsequent person-centred care theoretical works have been launched (Mitchell & Agnelli, 2015).

The central unifying assumption in Kitwood’s person-centred care is in relation to the concept of *personhood* and its preservation within the context of dementia. Kitwood believed that individuals with dementia are at risk for loss of their personhood based on a biomedical perspective of dementia that suggests parts of the self are lost as cognitive and functional impairments manifest. Drawing from a combination of transcendent, ethical and social discourses, he defined personhood as ‘a standing or status that is bestowed upon one human being by others, in the context of relationship and social being’ (Kitwood, 1997, p. 8). Thus, the second major assumption of Kitwood’s philosophy is that recognition and respect for personhood takes place within the context of social relationships and, as such, he establishes a linkage to Buber’s I-It/I-Thou philosophy (Buber, 1958). As such, if we depersonalize an individual with dementia by viewing them as a partial-person and a product of their condition, this propagates an I-It relationship in which a person is not considered a whole being, and a fulsome satisfying relationship is not possible. Conversely, I-Thou relations provide the pathway to realizing joy and fulfilment through human relationships where each person is valued as a unique and whole individual. It is from this relational perspective that Kitwood believed personhood must be viewed to understand dementia and care (Kitwood, 1997). A further assumption of Kitwood’s work involved the negative influence of *malignant social psychology*, referring to actions and attitudes of other people that function to undermine personhood. Alternately, to enhance personhood, he described *positive person work* that highlights various types of *affirming interactions* that aim to promote positive feelings, provide healing or nurture ability. Five of these indicators, recognition, negotiation, validation, facilitation and collaboration are specifically applicable to person-centred communication. Furthering Kitwood’s original work, Brooker (2004; 2007) provided additional conceptual clarification and expressed the major elements of the philosophy via the VIPS acronym: Value the person, enact an Individualized approach, understand the Perspective of the person with dementia and provide a Supportive social environment.

The desired person-centred care outcomes of Kitwood’s philosophy have individual-, provider- and system-level impacts. For the individual with dementia, it is hypothesized that their personal well-being is enhanced, and psychosocial needs are addressed when they are treated as a unique and whole individual who can fulfil social roles and engage in relationships with others. Thus, person-centred outcomes in dementia care include meeting physical/clinical needs as well as relational ones (Kitwood, 1997). Within Kitwood’s approach, the care providers (formal and informal) are also valued and respected as people with unique needs and feelings (Brooker, 2004; 2007) and, additionally may benefit from the richness of an open, accepting relationship that is borne from human interconnectedness. From a system-level perspective, Kitwood’s person-centred care promotes a cultural transformation away from a paradigm that is disease-oriented and fragmented to one that is collaborative, relationship-focused and encompasses the entirety of a person’s needs and preferences (American Geriatric Society, 2016; McCance, McCormack, & Dewing, 2011).

Kitwood’s work has also been used to support strategies to enhance person-centred communication outcomes between providers and with persons with dementia. In a study employing discourse analysis of conversations between long-term care residents with dementia and health care aides, Ryan and colleagues (2005) aimed to identify the communication and language strategies used during positive care interactions as defined by Kitwood. Four of Kitwood’s affirming interactions were chosen based on their applicability to communication with people living with dementia: recognition, negotiation, validation and facilitation, the latter also encompassing collaboration. Ryan and colleagues’ qualitative analysis (2005) found that these four strategies were evident in providers’ positive interactions with residents. They concluded that these strategies have the potential to improve meaningful interaction in long-term care by implementing a communication approach based on the enhancement of personhood (Ryan et al., 2005). Savundranayagam and Moore-Nielsen (2015) also found evidence of care providers’ use of recognition, negotiation, validation and facilitation person-centred communication strategies during conversations with long-term care home residents. They concluded that long-term care home staff need further training to use more diverse communication strategies that support personhood of people with dementia (Savundranayagam & Moore-Nielsen, 2015).

In summary, the theoretical literature base pertaining to person-centred care has grown exponentially over the past two decades, enabling refinement of the ability to define, implement and measure the outcomes of person-centred approaches in dementia care. However, this body of literature does not offer the fulsome detail that is needed to ground instrumental person-centred communication strategies in long-term care home/dementia care settings; thus, attention is now turned to the theoretical literature that applies to person-centred communication.

### Theoretical perspectives in person-centred communication

In consideration of extant theory that have been used to study person-centred communication, three theoretical perspectives emerged. A summary of the assumptions, key elements and outcomes of these perspectives is presented in Table 3. Upon critical review of the theories utilized to study person-centred communication within the healthcare context to date, a comprehensive theoretical approach that can sufficiently and concurrently address all elements of person-centred communication within the context of long-term care homes/dementia care is lacking. To illustrate, each of the theoretical perspectives will be examined from this viewpoint.

**Table 3.** Summary person-centred communication theoretical perspectives.

Author(s) Date Location	Theoretical perspective	Assumptions/Key elements	Outcomes
Coupland et al., 1988 Linguistics/Language Studies UK	Communication accommodation theory	•People modify communication based on their perception and belief of the other’s communication abilities •We tend to accommodate people we respect/admire and non-accommodate those we dislike or wish to diverge from; can manifest as either under-/over-accommodation •Both occur via five socio-linguistic strategies: approximation, interpretability, control, discourse management, emotional expression	•Overall: Positive interpersonal and intergroup interactions; resolution of conflict •Individual: improved healthcare outcomes and psychological well-being •Relational: Formation of equal role relations and rapport •In dementia/older adult care oProvider - less patronizing speech, improved emotional tone oResidents - increased interaction •System/society: Reduction of negative ageing stereotypes
Ryan et al., 1986 Ageing Psychology Canada	Communication Predicaments of Ageing Model	•Providers often accommodate speech when conversing with older adults based on distorted perspectives of dependence and incompetency •Older adults experience communication predicaments – discrepancies between perceived and actual abilities •Depicted as a cyclical process oEncounter with an older adult oRecognition of cues oStereotyped expectations oModified speech oNegative outcomes for both parties oReinforced cues/views	•Individual: Reduction or restraint of communication opportunities, reduced self-esteem, reduced participation in activities •Person with dementia: increased dependency; loss of communication skills/abilities •Care provider: Reinforcement of stereotypical behaviour and views •Relational: Lack of respect and jeopardizes opportunity to form authentic, caring relationships •System/society: Reinforces negative ageing stereotypes
Ryan et al., 1995 Ageing Psychology Canada	Communication Enhancement Model	•Addresses communication predicaments of ageing and negative outcomes through creating awareness (provider), creating higher expectations (older adult) and recognition of supportive environment •Action-oriented health promotion approach for change at individual and system levels •Cyclical process oEncounter with an older adult oRecognition of individual cues oModified communication to accommodate needs oIndividualized assessment, and oResultant positive communication outcomes oEnhanced knowledge/awareness for next encounter	•Overall: communication climate that is empowering and satisfying for both provider and older adult •Individual: empowerment, enhanced health/well-being, maximized communication competence and opportunities •Provider: improved communication knowledge and skills, improved assessment and intervention, empowerment, increased work satisfaction •Relational: improved quality of relationships •System: modify environment to support provider-older adult communication

#### Communication accommodation theory

In consideration of Coupland and colleagues’ work (1988), the Communication Accommodation Theory assumes that communicators modify or adjust their speech and non-verbal behaviours to each other based on individual values, perceptions and motivations. In other words, speakers *accommodate* their approaches based on *their belief* of the other persons’ communicative capabilities (Coupland et al., 1988). Due to its dual socio-linguistic viewpoint, this theoretical perspective has the potential to support both effective linguistic accommodation strategies and relational interactions required within person-centred communication. However, it lacks sufficient detail to distinguish between specific forms and strategies of linguistic and psychological accommodation (Farzadnia & Giles, 2015), particularly in the context of dementia.

#### Communication predicaments of ageing model

Inspired by the Communication Accommodation Theory, this model (Ryan et al., 1986) asserted that the natural tendency for health care providers to accommodate their speech when conversing with older adults is based on distorted perspectives of dependency and incompetence. Although this model creates a mechanism to identify and create awareness of the communication predicaments associated with ageing, it does not provide theoretical or practical guidance as to how to break or reverse the cycle. It also neglects to address the broader environment or contextual considerations in which interactions and relationships occur; thus, does not offer insight regarding impacts on system- or policy-level changes to promote person-centred dementia communication.

#### Communication enhancement model

The Communication Enhancement Model (Ryan et al., 1995) was introduced to address the communication predicaments and negative health outcomes outlined in the Communication Predicaments of Ageing Model. Embedded within an action-oriented health promotion framework, the Communication Enhancement Model aims to direct change at both the individual and system levels (Ryan et al., 1995). The model has also been positioned to provide a theoretical basis for enhancing communication strategies between providers and people with dementia (Orange, et al., 1995). Establishing a link to person-centred dementia care and communication, the model promotes individualized assessment and knowledge of the person and their strengths. Therefore, individuals with cognitive impairment and diminished verbal communication abilities can also experience the benefits of positive, meaningful interactions (Orange et al., 1995).

In summary, the Communication Enhancement Model appears to be best positioned to underpin empirical study of care provider–resident communication research in long-term care homes and dementia care. The model has potential to support successful linguistic, relational and social aspects of person-centred dementia communication within a context of a supportive environment. This is evidenced in grounded theory research conducted in long-term care homes. Wolf (2017) found that when health care aides perceived the resident as a respected person with whom they had a relationship, they used communication enhancement strategies to meet individual physical and psychosocial needs. The only limitation noted is that the specific relational aspects of person-centred communication are not overtly evident in the Communication Enhancement Model which would help guide the measurement and interpretation of communication interactions and outcomes. However, this limitation could be addressed by integrating the affirming interactions of Kitwood’s person-centred care that are associated with person-centred communication (i.e. recognition, negotiation, validation and facilitation/collaboration) into the Communication Enhancement Model.

### Overview of communication theoretical perspectives

The literature has seen growth and refinement of communication theoretical works relevant to person-centred communication. Earlier perspectives offered a lens to study effective interpersonal communication between individuals in a general sense. Subsequent developments have provided additional refinement to theoretical works, along with a focus on effective communication with older adults within care settings. These perspectives imbue a collective assumption that effective and respectful communication approaches are beneficial to both parties as they promote quality interpersonal relations and excellence in care. Conversely, ineffective and disrespectful communication can have negative individual consequences, such as loss of skills, abilities and confidence in relation to communication opportunities, as well as diminish the potential to build meaningful relationships.

Some of the theories share common outcomes including improved psychological well-being, increased opportunities for interaction, and empowerment at the individual level (Coupland et al., 1988; Ryan et al., 1995). Provider outcomes include increased knowledge and skills in communication (Ryan et al., 1995), a decrease in patronizing speech, improved emotional response (Coupland et al., 1988) and increased work satisfaction (Ryan et al., 1995). Shared system-level impacts include the creation of a positive communication climate and transformative efforts to reduce ageist views within workplaces and society (Coupland et al., 1988; Ryan et al., 1995).

These theoretical perspectives were not originally developed to study person-centred communication per se; therefore, some variation exists in consideration of their application to this area of research. For example the Communication Predicaments of Ageing Model appears best situated to support the linguistic (language-based) aspects of person-centred communication. The Communication Accommodation Theory and Communication Enhancement Model appear to best acknowledge the dual nature (i.e. linguistic and person-centred/relational elements) of person-centred communication. However, the specific linguistic and person-centred aspects of person-centred communication are not overtly evident in these theoretical works and require additional expansion to adequately support the study of person-centred communication in long-term care homes/dementia care. As such, the PC-CEM as described above, is offered to address these gaps and provide additional theoretical guidance within this field of research.

## Concluding thoughts

Perspectives from the genres of philosophy, psychology, nursing and linguistics have contributed to the present shape of the person-centred care and person-centred communication theoretical landscape. A collective assumption among the theoretical works relevant to this field is that the provision of quality physical and psychological care and service within long-term care home and dementia care contexts is enabled through effective interactions and meaningful relationships between providers and residents. Thus, to appreciate the intention of Martin Buber’s *real living* and realize fulsome relationships through communication that is person-centred, it is essential to embrace and foster interconnectedness and relationships between providers and those seeking care and services. To this end, the emergent Person-Centred Communication Enhancement Model offers a refinement and organization of existing person-centred and communication theoretical works that is necessary to advance our understanding of the intricacies of person-centred communication within the long-term care home and dementia care contexts.

## References

[bibr1-14713012221080252] American Geriatrics Society Expert Panel on Person-Centered Care (2016). Person-centered care: A definition and essential elements. Journal of the American Geriatrics Society, 64(1), 15–18. DOI: 10.1111/jgs.13866. 26626262

[bibr2-14713012221080252] Aubry F. Etheridge F. Couturier Y. (2013). Facilitating change among nursing assistants in long-term care. Online Journal of Issues in Nursing, 18(1). DOI: 10.3912/OJIN.Vol18No01PPT01. 23452195

[bibr3-14713012221080252] Barbosa A. Marques A. Sousa L. Nolan M. Figueiredo D. (2016). Effects of a psycho-educational intervention on direct care workers’ communicative behaviors with residents with dementia. Health Communication, 31(4), 453–459. DOI: 10.1080/10410236.2014.965382. 26400182

[bibr4-14713012221080252] Brooker D. (2004). What is person-centred care in dementia? Reviews in Clinical Gerontology, 13(3), 215–222. DOI: 10.1017/s095925980400108x.

[bibr5-14713012221080252] Brooker D. (2007). Person-centred dementia care: Making services better. London, UK: Jessica Kingsley Publishers. 10.7748/nop.19.5.22.s21 27726617

[bibr6-14713012221080252] Buber M. (1958). I and thou. New York: Scribner.

[bibr7-14713012221080252] Carpiano R. M. Daley D. M. (2006). A guide and glossary on postpositivist theory building for population health. Journal of Epidemiology and Community Health, 60(7), 564–570. DOI: 10.1136/jech.2004.031534. 16790824 PMC2566228

[bibr8-14713012221080252] Coleman C. K. Medvene L. J. Van Haitsma K. (2013). A person-centered care intervention for geriatric certified nursing assistants. Gerontologist, 53(4), 687–698. DOI: 10.1093/geront/gns135. 23114564

[bibr9-14713012221080252] Colquhoun H. L. Squires J. E. Kolehmainen N. Fraser C. Grimshaw J. M. (2017). Methods for designing interventions to change healthcare professionals’ behaviour: A systematic review. Implementation Science, 12(30), 30–11. DOI: 10.1186/s13012-017-0560-5. 28259168 PMC5336662

[bibr10-14713012221080252] Coupland N. Coupland J. Giles H. Henwood K. (1988). Accommodating the elderly: Invoking and extending a theory. Language in Society, 17(1), 1–41. DOI: 10.1017/S0047404500012574.

[bibr11-14713012221080252] Custers A. F. J. Kuin Y. Riksen-Walraven M. Westerhof G. J. (2011). Need-support and well-being during morning care activities: An observational study on resident-staff interactions in nursing homes. Aging & Society, 31(8), 1425–1442. DOI: 10.1017/s0144686x10001522.

[bibr12-14713012221080252] Davis M. H. (1994). Empathy: A social psychological approach. Boulder, CO: Westview Press.

[bibr13-14713012221080252] Dewar B. Nolan M. (2013). Caring about caring: Developing a model to implement compassionate relationship-centred care in an older people care setting. International Journal of Nursing Studies, 50(9), 1247–1258. DOI: 10.1016/j.ijnurstu.2013.01.008. 23427893

[bibr14-14713012221080252] Downs M. Collins L. (2015). Person-centred communication in dementia care. Nursing Standard, 30(11), 37–41. DOI: 10.7748/ns.30.11.3710.7748/ns.30.11.37.s45. 26554996

[bibr15-14713012221080252] Duggan P. S. Geller G. Cooper L. A. Beach M. C. (2006). The moral nature of patient-centredness: Is it “just the right thing to do?” Patient Education and Counseling, 62(2), 271–276. DOI: 10.1016/j.pec.2005.08.001. 16356677

[bibr16-14713012221080252] Ennis L. Mansell W. McEvoy P. Tai S. (2019). A systematic scoping review and synthesis of dementia and communication theory. Dementia, 18(6), 2261–2281. DOI: 10.1177/1471301217744069. 29216743

[bibr17-14713012221080252] Farzadnia S. Giles H. (2015). Patient-provider health interactions: A communication accommodation theory perspective. International Journal of Society, Culture & Language, 3(2), 17–34.

[bibr18-14713012221080252] Green H. E. (2014). Use of theoretical and conceptual frameworks in qualitative research. Nurse Researcher, 21(6), 34–38. DOI: 10.7748/nr.21.6.34.e1252. 25059086

[bibr19-14713012221080252] Grosch K. Medvene L. Wolcott H. (2008). Person-centered caregiving instruction for geriatric nursing assistant students. Journal of Gerontological Nursing, 34(8), 23–31. DOI: 10.3928/00989134-20080801-07. 18714603

[bibr20-14713012221080252] Harding E. Wait S. Scrutton J. (2015) The state of play in person-centred care: A pragmatic review of how person-centred care is defined, applied and measured (139). London: Health Policy Partnership. Retrieved from https://www.healthpolicypartnership.com/app/uploads/The-state-of-play-in-person-centred-care.pdf.

[bibr21-14713012221080252] James D. M. Hall A. Lombardo C. McGovern W. (2016). A video feedback intervention for workforce development: Exploring staff perspective using longitudinal qualitative methodology. Journal of Applied Research in Intellectual Disabilities, 29(2), 111–123. DOI: 10.1111/jar.12161. 25772003

[bibr22-14713012221080252] James D. M. Hall A. Phillipson J. McCrossan G. Falck C. (2013). Creating a person-centred culture within the North East Autism Society: Preliminary findings. British Journal of Learning Disabilities, 41(4), 296–303. DOI: 10.1111/j.1468-3156.2012.00757.x.

[bibr23-14713012221080252] Kagan A. Simmons‐Mackie N. Rowland A. Huijbregts M. Shumway E. Mcewen S. Threats T. Sharp S. (2008). Counting what counts: A framework for capturing real-life outcomes of aphasia intervention. Aphasiology, 22(3), 258–280. DOI: 10.1080/02687030701282595.

[bibr24-14713012221080252] Kitwood T. (1993). Towards a theory of dementia care: The interpersonal process. Ageing and Society, 13(1), 51–67. DOI: 10.1017/S0144686X00000647. 11654434

[bibr25-14713012221080252] Kitwood T. (1997). Dementia reconsidered: The person comes first. Buckingham: Open University Press.

[bibr26-14713012221080252] Kitwood T. Bredin K. (1992). Towards a theory of dementia care: Personhood and well-being. Ageing and Society, 12, 269–287. DOI: 10.1017/S014468X0000502X. 11654434

[bibr27-14713012221080252] Li J. Porock D. (2014). Resident outcomes of person-centered care in long-term care: A narrative review of interventional research. International Journal of Nursing Studies, 51(10), 1395–1415. DOI: 10.1016/j.ijnurstu.2014.04.003. 24815772

[bibr28-14713012221080252] Lobchuk M. Halas G. West C. Harder N. Tursunova Z. Ramraj C. (2016). Development of a novel empathy-related video-feedback intervention to improve empathic accuracy of nursing students: A pilot study. Nurse Education Today, 46, 86–93. DOI: 10.1016/j.nedt.2016.08.034. 27614549

[bibr29-14713012221080252] Lobchuk M. Hoplock L. Halas G. West C. Dika C. Schroeder W. Ashcroft T. Clouston K. C. Lemoine J. (2018). Heart health whispering: A randomized, controlled pilot study to promote nursing student perspective-taking on carers’ health risk behaviors. BMC Nursing, 17, 21. DOI: 10.1186/s12912-018-0291-1. 29849504 PMC5968556

[bibr30-14713012221080252] McCance T. McCormack B. Dewing J. (2011). An exploration of person-centredness in practice. OJIN: Online Journal of Issues in Nursing, 16(2), 1. DOI: 10.3912/ojin.vol16no02man01. Retrieved from http://www.nursingworld.org/MainMenuCategories/ANAMarketplace/ANAPeriodicals/OJIN/TableofContents/Vol-16-2011/No2-May-2011/Person-Centredness-in-Practice.html. 22088150

[bibr32-14713012221080252] McCormack B. McCance T. V. (2006). Development of a framework for person-centred nursing. Journal of Advanced Nursing, 56(5), 472–479. DOI: 10.1111/j.1365-2648.2006.04042.x. 17078823

[bibr33-14713012221080252] McGilton K. S. Boscart V. Fox M. Sidani S. Rochon E. Sorin-Peters R. (2009). A systematic review of the effectiveness of communication interventions for health care providers caring for patients in residential care settings. Worldviews on Evidence-Based Nursing, 6(3), 149–159. DOI: 10.1111/j.1741-6787.2009.00155.x. Retrieved from http://ovidsp.ovid.com/ovidweb.cgi?T=JS&PAGE=reference&D=emed9&NEWS=N&AN=2009480762%5Cnhttp://ovidsp.ovid.com/ovidweb.cgi?T=JS&PAGE=reference&D=psyc6&NEWS=N&AN=2009-14393-003 19523033

[bibr34-14713012221080252] McGilton K. S. Heath H. Chu C. H. Boström A. M. Mueller C. Boscart V. M. McKenzie-Green B. Moghabghab R. Bowers B. (2012). Moving the agenda forward: A person-centred framework in long-term care. International Journal of Older People Nursing, 7(4), 303–309. DOI: 10.1111/opn.12010. Retrieved from http://ovidsp.ovid.com/ovidweb.cgi?T=JS&CSC=Y&NEWS=N&PAGE=fulltext&D=ovftn&AN=01253235-201212000-00009. 23164252

[bibr35-14713012221080252] McGilton K. S. Rochon E. Sidani S. Shaw A. Ben-David B. M. Saragosa M. Boscart V. M. Wilson R. Galimidi-Epstein K. K. Pichora-Fuller M. K. (2016). Can we help care providers communicate more effectively with persons having dementia living in long-term care homes? American Journal of Alzheimer's Disease and Other Dementias, 32(1), 41–50. DOI: 10.1177/1533317516680899. PMC5302128 27899433

[bibr36-14713012221080252] McGilton K. S. Sorin-Peters R. Rochon E. Boscart V. Fox M. Chu C. H. Stewart S. C. Sidani S. (2018). The effects of an interprofessional patient-centered communication intervention for patients with communication disorders. Applied Nursing Research, 39(November), 189–194. DOI: 10.1016/j.apnr.2017.11.017. 29422157

[bibr37-14713012221080252] McGilton K. Sorin-Peters R. Sidani S. Rochon E. Boscart V. Fox M. (2010). Focus on communication: Increasing the opportunity for successful staff-patient interactions. International Journal of Older People Nursing, 6(1), 13–24. DOI: 10.1111/j.1748-3743.2010.00210.x. 21303460

[bibr38-14713012221080252] Mitchell G. Agnelli J. (2015). Person-centred care for people with dementia: Kitwood reconsidered. Nursing Standard, 30(7), 46–50. DOI: 10.7748/ns.30.7.46.s47. 26463810

[bibr39-14713012221080252] Nolan M. Davies S. Brown J. (2006). Transitions in care homes: Towards relationship-centred care using the “Senses Framework”. Quality in Ageing and Older Adults, 7(3), 5–14. DOI: 10.1108/14717794200600015.

[bibr40-14713012221080252] O’Connell B. Ostaszkiewicz J. Sukkar K. Plymat K. (2008). The tri-focal model of care: Advancing the teaching-nursing home concept. International Journal of Nursing Practice, 14(6), 411–417. DOI: 10.1111/j.1440-172X.2008.00714.x. 19126068

[bibr41-14713012221080252] Orange J. B. Ryan E. B. Meredith S. D. MacLean M. J. (1995). Application of the communication enhancement model for long-term care residents with Alzheimer’s disease. Topics in Language Disorders, 15(2), 20-35. DOI: 10.1097/00011363-199502000-00004.

[bibr57-14713012221080252] Passalacqua S. A. Harwood J. (2012). VIPS communication skills training for paraprofessional dementia caregivers: An intervention to increase person-centered dementia care. Clinical Gerontologist, 35(5), 425–445 21p. DOI: 10.1080/07317115.2012.702655

[bibr42-14713012221080252] Røsvik J. Kirkevold M. Engedal K. Brooker D. Kirkevold Ø. (2011). A model for using the VIPS framework for person-centred care for persons with dementia in nursing homes: A qualitative evaluative study. International Journal of Older People Nursing, 6(3), 227–236. DOI: 10.1111/j.1748-3743.2011.00290.x. 21884488

[bibr43-14713012221080252] Ryan E. B. Byrne K. Spykerman H. Orange J. B. (2005). Evidencing Kitwood’s personhood strategies: Conversation as care in dementia. In Boyd B.H. (Ed), Alzheimer talk, text and context: Enhancing communication (pp. 18–36). New York: Palgrave MacMillan. DOI: 10.1057/9780230502024_2.

[bibr44-14713012221080252] Ryan E. B. Giles H. Bartolucci G. Henwood K. (1986). Psycholinguistic and social psychological components of communication by and with the elderly. Language and Communication, 6(1–2), 1–24. DOI: 10.1016/0271-5309(86)90002-9.

[bibr45-14713012221080252] Ryan E. B. Meredith S. D. MacLean M. J. Orange J. B. (1995). Changing the way we talk with elders: Promoting health using the communication enhancement model. International Journal of Aging and Human Development, 41(2), 89–107. DOI: 10.2190/FP05-FM8V-0Y9F-53FX. 8550234

[bibr46-14713012221080252] Sabat S. R. (2002). Surviving manifestations of selfhood in Alzheimer’s disease. Dementia, 1(1), 25–36. DOI: 10.1177/147130120200100101.

[bibr47-14713012221080252] Sabat S. R. Lee J. M. (2011). Relatedness among people diagnosed with dementia: Social cognition and the possibility of friendship. Dementia, 11(3), 315–327. DOI: 10.1177/1471301211421069.

[bibr48-14713012221080252] Savundranayagam M. Y. Moore-Nielsen K. (2015). Language-based communication strategies that support person-centered communication with persons with dementia. International Psychogeriatrics, 27(10), 1707–1718. DOI: 10.1017/S1041610215000903. 26334515

[bibr49-14713012221080252] Savundranayagam M. Y. Sibalija J. Scotchmer E. (2016). Resident reactions to person-centered communication by long-term care staff. American Journal of Alzheimer's Disease and Other Dementias, 31(6), 530–537. DOI: 10.1177/1533317515622291. PMC10852976 26744507

[bibr50-14713012221080252] Small J. Perry J. A. (2012). Training family care partners to communicate effectively with persons with Alzheimer’s disease: The TRACED program. Canadian Journal of Speech-Language Pathology & Audiology, 36(4), 332–350.

[bibr51-14713012221080252] Squires J. E. Hoben M. Linklater S. Carleton H. L. Graham N. Estabrooks C. A. (2015). Job satisfaction among care aides in residential long-term care: A systematic review of contributing factors, both individual and organizational. Nursing Practice and Research, 2015, 157924. DOI: 10.1155/2015/157924. PMC4541006 26345545

[bibr52-14713012221080252] Viau-Guay A. Bellemare M. Feillou I. Trudel L. Desrosiers J. Robitaille M. J. (2013). Person-centered care training in long-term care settings: Usefulness and facility of transfer into practice. Canadian Journal on Aging, 32(1), 57–72. DOI: 10.1017/S0714980812000426. 23339880

[bibr54-14713012221080252] Williams K. N. Herman R. Gajewski B. Wilson K. (2009). Elderspeak communication: Impact on dementia care. American Journal of Alzheimer's Disease and Other Dementias, 24(18), 11–20. DOI: 10.1016/j.jacc.2007.01.07610.1177/1533317508318472. PMC2823803 18591210

[bibr55-14713012221080252] Williams K. N. Perkhounkova Y. Bossen A. Hein M. (2016). Nursing home staff intentions for learned communication skills: Knowledge to practice. Journal of Gerontological Nursing, 42(3), 26-34. DOI: 10.3928/00989134-20160212-06. 26934971 PMC4841447

[bibr56-14713012221080252] Wolf L. (2017). Communication interactions of health care aides with individuals with dementia (Doctoral dissertation). Winnipeg, Manitoba, Canada: University of Manitoba. Retrieved from https://mspace.lib.umanitoba.ca/bitstream/handle/1993/32233/wolf_lynda.pdf?sequence=1.

